# Insights on Droplet Digital PCR–Based Cellular Kinetics and Biodistribution Assay Support for CAR-T Cell Therapy

**DOI:** 10.1208/s12248-021-00560-6

**Published:** 2021-03-02

**Authors:** Hiroshi Sugimoto, Susan Chen, Jean-Pierre Minembe, Johara Chouitar, Xingyue He, Haiqing Wang, Xiaodong Fang, Mark G. Qian

**Affiliations:** 1 Department of Drug Metabolism and Pharmacokinetics, Takeda Pharmaceuticals International Co, 125 Binney Street, Cambridge, Massachusetts 02142 USA; 2 Department of Drug Metabolism and Pharmacokinetics, Takeda Pharmaceuticals International Co, 35 Landsdowne Street, Cambridge, Massachusetts 02139 USA; 3 Department of Immuno Oncology DDU, Takeda Pharmaceuticals International Co, 40 Landsdowne Street, Cambridge, Massachusetts 02139 USA

**Keywords:** Automated DNA extraction, Biodistribution, Cellular kinetics, Droplet digital PCR, gDNA recovery

## Abstract

Characterizing *in vivo* cellular kinetics and biodistribution of chimeric antigen receptor T (CAR-T) cells is critical for toxicity assessment, nonclinical and clinical efficacy studies. To date, the standardized assay to characterize CAR-T cell distribution, expansion, contraction, and persistence profiles is not readily available. To overcome this limitation and increase comparability among studies, we have established a universal protocol for analysis. We established a duplexing ddPCR protocol for the CAR-T transgene and reference gene to normalize the genomic DNA input prepared from mouse blood and tissues. The high-throughput gDNA extraction method enabled highly reproducible gDNA extraction while eliminating labor-intensive steps. The investigational CAR-T cells were intravenously injected into immunodeficient mice bearing human colorectal cancer xenografts. The blood and tissue samples were collected to measure the cellular kinetics by ddPCR and flow cytometry. The standard curves were linear throughout the calibration range with acceptable intra- and inter-day precision and accuracy. The gDNA recovery study performed by spiking in the exo-gene plasmid DNA or CAR-T cells revealed that the recovery ranged from 60 to 100% in blood and tissue homogenates. The use of both units of copy/μg gDNA and copy/μL blood met the current regulatory requirement and allowed for a systematic understanding of CAR-T cell expansion and a direct comparison with the flow cytometry data. A standardized ddPCR assay, including automated gDNA extraction procedures, has been established for evaluating cellular kinetics and biodistribution in CAR-T cell therapies.

**Supplementary Information:**

The online version contains supplementary material available at 10.1208/s12248-021-00560-6.

## INTRODUCTION

### CAR-T Background Information, Opportunity, and Challenge

The unprecedented positive clinical response of chimeric antigen receptor T (CAR-T) cell therapy for CD19-positive aggressive B cell lymphoma and B cell precursor acute lymphoblastic leukemia brought the adoptive cell immunotherapy into the mainstream of global hematologic cancer treatment (tisagenlecleucel, Kymriah®, Novartis and axicabtagene ciloleucel, Yescarta®, Gilead (1)). The recent pioneering clinical pharmacology and cellular kinetics research in B cell acute lymphoblastic leukemia have revealed that the higher expansion of tisagenlecleucel in blood correlated with the number of complete responding patients and the severity of cytokine release syndrome (2, 3). The opportunities and challenges of CAR-T cell therapy remain in the solid tumor space due to the tumor-associated factors (lack of specific solid tumor-associated antigen, its expression level, and heterogeneity), T cell–associated factors (T cell exhaustion and lack of persistence), and host-associated factors (immunosuppressive tumor micro-environment and insufficient T cell infiltration) (4). Therefore, a comprehensive understanding of the cellular kinetics and biodistribution of CAR-T cells, especially for the targeted tissue such as a tumor, is critical.

### Cellular Kinetics and Biodistribution Study

Although the conventional absorption/distribution/metabolism/elimination (ADME) studies are usually not relevant for CAR-T cell therapy, the cellular kinetics, biodistribution, and persistence of CAR-T cells, as well as the level of the transgene production in the target and non-targeted tissues, need to be evaluated (5). The kinetics of CAR-T cell therapy is intricate and influenced by physiological functions of the targeted cell type and the abundance of CAR targeting antigen (6). Recently, Mueller *et al*. reported that the tisagenlecleucel level transiently declined right after the administration and rapidly showed a multi-log expansion from the baseline to reach the maximal expansion followed by a multiexponential decline during the next few months in human blood (2). When it comes to the cellular kinetics and biodistribution study of CAR-T cells in nonclinical studies, there are a very limited number of published studies available. Nonclinical cellular kinetics and biodistribution should be designed to support the dose selection, administration route, and dosing schedule for clinical trials combined with the previous clinical research outcome for the related CAR-T cells products.

### Technology for Cellular Kinetics and Biodistribution

Several bioanalytical methods are available to detect and quantify CAR-T cells in biomatrices, i.e., quantitative polymerase chain reaction (qPCR), droplet digital PCR (ddPCR), flow cytometry, fluorescence, and radiolabeled imaging. The current gold standard to measure the cellular kinetics is a qPCR-based assay to detect the transgene specific to CAR-T cells. The FDA guidance recommended that the assay should have a demonstrated limit of quantification (LOQ) of ≤50 copies of product per 1 μg genomic DNA (gDNA) (7). Muller *et al*. demonstrated the same quantification limit in the qPCR-based assay for detecting the tisagenlecleucel transgene in peripheral blood and bone marrow samples in a clinic (8). However, the assay standardization of cellular kinetics and biodistribution has not reached a consensus among the regulatory agency, pharmaceutical industry, and academic community to support the cell therapy program. Digital PCR enables absolute quantification by a combination of limiting dilutions, end-point PCR, and Poisson statistics (9). Hindson *et al*. introduced the high-throughput water-oil emulsion droplet technology–based digital PCR system, which enabled sensitive, selective, and reproducible detection of rare alleles and the absolute quantification of targeted gene copy numbers (10). However, ddPCR for characterizing *in vivo* cellular kinetics and biodistribution of CAR-T cell therapy is still in its infancy stage (11, 12). The transgene copy number normalized by the associated amount of gDNA (copy/μg gDNA) is the conventional unit to normalize the gDNA extraction variability during the sample preparation process (7). However, if CAR-T cells expand significantly and their gDNA dominate the gDNA extracted from blood samples after the administration *in vivo*, the apparent transgene copy number with the copy/μg gDNA unit will underestimate the actual CAR-T cell expansion (13). The transgene copy number normalized by the associated blood volume or tissue weight (copy/μL blood or mg tissue) may circumvent the drawback of the copy/μg gDNA unit since the copy/μL blood unit is not affected by the individual difference of gDNA content in blood, especially during the treatment of lymphodepleting chemotherapy to improve CAR-T cell persistence and a lower risk of CD19^+^ relapse (14). Besides, the data generated with the copy/μL blood unit is useful for comparing with the data generated by flow cytometry, which detects the cell surface expression of CAR-T cells in the blood (12, 15). To our best knowledge, there is no standardized ddPCR-based cellular kinetics and biodistribution assay available with the current regulatory compliant unit (copy/μg gDNA) as well as the pharmacologically meaningful unit (copy/μL blood or copy/mg tissue). In this report, we propose a standardized ddPCR assay including automated gDNA extraction procedures for evaluating cellular kinetics and biodistribution in CAR-T cell therapies.

## MATERIALS AND METHODS

### Chemicals and Reagents

The reference material of the investigational CAR-T construct was prepared in-house (4.3 μg/μL) with a proprietary target binder. Primers and TaqMan probes used for droplet digital PCR assay were purchased from Integrated DNA Technologies, Inc. (Coralville, IA). The yeast HIS3 and LEU2 plasmid DNA were obtained from Aldevron, LLC (Fargo, ND). Bio-Rad QX200 AutoDG Droplet Digital PCR System™ including automated droplet generator, C1000 Touch™ Thermal Cycler, and QX200 droplet reader and their consumables were from Bio-Rad Laboratories (Hercules, CA). DNeasy Blood & Tissue Kit is from Qiagen (Valencia, CA). KingFisher magnetic beads processor™, MagMAX™ DNA Multi-Sample Ultra 2.0 Kit, and Ambion™ RNase A were from Thermo Fisher Scientific (Waltham, MA). GentleMACS Octo Dissociator was from Miltenyi Biotec (Somerville, MA).

### *In Vivo* Human-Derived Xenograft Tumor Studies in Mice

All animal research and veterinary care were performed under the Guide for the Care and Use of Laboratory Animals under approved protocols of the Takeda Boston Institutional Animal Care and Use Committee in a facility accredited by the Association for Assessment and Accreditation of Laboratory Animal Care International (AAALAC). A suspension of the human colorectal cancer model cell line was subcutaneously injected into the flank of female NOD SCID Gamma (NSG) mice at 2.0 × 10^6^ cells/mouse. The projected tumor volume at the initial date is approximately 150 mm^3^. The animals were sorted into treatment groups (*n* = 3) and were given a single intravenous administration of CAR-T positive cells at a dose of 1.0 × 10^6^ CAR positive T cells/100 μL/mouse. Mice were sacrificed at designated time points (1 h, day 1, 3, 7, 10, 14, 21, and 28) after the administration, then blood and tumor samples were harvested. Tumor volume and body weight were measured before the sample collection. Samples were frozen and stored under − 80 °C until sample analysis.

### Extraction of gDNA from Mouse Blood and Tissue Samples

gDNA extraction from mouse blood and tissue homogenate was conducted with MagMAX™ DNA Multi-Sample Ultra 2.0 Kit with minor modifications. The gDNA was also extracted from mouse blood and tissue samples using MagMAX™ DNA Multi-Sample Ultra 2.0 Kit in the presence of Proteinase K. Ambion™ RNase A (5 μL each, affinity purified, Thermo Fisher Scientific) was added to make sure the contamination of RNA is negligible. Tissue samples were homogenized in nine volumes of PBS containing 15 mM EDTA with gentleMACS Octo Dissociator in the mode of RNA_01_01. Binding enhancer solution (10 or 40 μL), homogenate solution (100 or 400 μL), and proteinase K (10 or 40 μL) were mixed well in a plate and then incubated the plate at 65 °C overnight. The DNA extraction process was automated with the KingFisher™ Flex system using the sample processing programs (MagMAX_Ultra2_200μL_Flex and MagMAX_Ultra 2_Tissue_V) according to the manufacture’s protocol. The gDNA purity from each extracted sample was monitored by NanoDrop 2000 spectrophotometer using the absorbance ratio at 260 and 280 nm (A_260_/A_280_).

### Determination of Transgene Copy Number in CAR-T Cells by Droplet Digital PCR

The TaqMan PCR reaction mixture consisted of 2× ddPCR supermix for probes (No dUTP), 20× primer and probes (detailed information described in Supplemental Table I), 40× restriction enzyme (XbaI, EcoRI-HF, 10 U/μL, New England Biolabs, Ipswich, MA), and template (4 μL) in a final volume of 20 μL. The droplets were generated, emulsified, and transferred to a 96-well PCR plate by QX200™ Droplet Generator. The head-sealed plate was placed on the C1000 Touch™ Thermal Cycler and the target-specific gene was amplified as follows: The thermal cycling condition was 95 °C for 5 min (1 cycle), 40 cycles at 95 °C for 0.5 min, and 63 °C for 2 min, and hold at 4 °C. After the thermal cycling, the positive and negative fluorescent droplets were counted in the QX100 Droplet Reader. The genomic copy numbers were calculated with the Quanta Soft program (Bio-Rad, Hercules, CA). Quantitative droplet digital PCR was performed on 200 ng of gDNA per reaction as a maximum input. The CD28 co-stimulatory and CD3ζ signal transduction domain-specific primers and probe were synthesized to amplify the junction between CD28 and CD3ζ. The distribution of the transgene target sequence in the droplet partitions is described by Poisson distribution. The absolute quantification of the transgene target was based on the ratio of positive signal to all droplet partitions at the end of PCR reaction as follows. The droplet volume (V_droplet_) was 0.85 nL as a defined parameter to calculate the copy number concentration. The data were analyzed with QuantaSoft analysis software version 1.7 (Bio-Rad).

$$ \mathrm{Concentration}=-\frac{\ln \left(\frac{\mathrm{Number}\ \mathrm{of}\ \mathrm{negative}\ \mathrm{droplets}}{\mathrm{Number}\ \mathrm{of}\ \mathrm{total}\ \mathrm{droplets}}\right)}{{\mathrm{V}}_{\mathrm{droplet}}} $$

### Assay Performance for ddPCR for Cellular Kinetics Assays

The ddPCR assay performance was evaluated based on a fit-for-purpose approach for selectivity, linearity, intra- and inter-assay precision, accuracy, and robustness. The selectivity of the ddPCR assay was evaluated by the no template control (NTC, water) to monitor potential contamination and primer-dimer formation, which could cause false-positive results. A linear model fitted by least-squares linear regression with weighting factor 1/*x*^2^ was used to describe the calibration curve. The calibration curve and quality control (QC) samples were prepared in 50 μg/mL of gDNA solution extracted from mouse blood. The concentration of the upper limit of quantification (ULOQ) sample was set at 3460 copies/μL reaction, which is below the upper detection limit (5000 copies/μL reaction). The ULOQ sample was serially diluted at dilution ratios of 0.0003, 0.001, 0.003, 0.01, 0.03, 0.1, and 0.3 to prepare the standard curve in 50 μg/mL of gDNA solution. The QC samples were prepared at the dilution ratios of 0.0008 (lower limit of quantitation, LLQC), 0.008 (low-QC, LQC), 0.08 (medium-QC, MQC), and 0.8 (high-QC, HQC), respectively, in 50 μg/mL of gDNA solution. The plasmid was linearized by the restriction enzyme (XbaI+ EcoRI-HF) to facilitate the PCR amplification efficiency. The content of the PCR reaction mixture is summarized as follows. A 20 μL aliquot of the TaqMan PCR reaction mixture was used for the droplet generation using QX200 AutoDG Droplet Digital PCR System.

**Table:** 

The PCR reaction mixture (per reaction)	
2x ddPCR Supermix for Probes (No dUTP)	11 μL
Primer [2 μmol/L (final conc. 100 nmol/L)]	1.1 μL
Probe [2 μmol/L (final conc. 100 nmol/L)]	1.1 μL
Restriction enzyme mix 10 U/μL	0.55 μL
H_2_O	3.85 μL
CAR-T transgene plasmid or QC sample	4.4 μL
Total	22 μL

The assay robustness was evaluated by the repeatability, including intra- and inter-assay precision and accuracy. The relative error (%RE) criteria of the back-calculated concentrations to the nominal concentrations were set within ±20% of nominal values (for LLOQ; ±25%). The sample stability was tested in incurred mouse blood collected after a single intravenous administration of CAR-T positive cells at a dose of 1.0 × 10^6^ cells/mouse and stored in a freezer set at −80 °C for 2 months.

### Flow Cytometry Analysis

Isolation of immune cells from fresh blood was conducted by adding 1 mL ACK lysis buffer per 100 μL of blood, and lysis was performed on ice for 5 min. The lymphocyte fraction was depleted of red blood cells by the lysis. The cells were resuspended in the staining buffer (Thermo Fisher Scientific) and counted, then labeled with the viability stain (Zombie Aqua, BioLegend) in PBS for 15 min at room temperature, washed, and incubated with anti-human CD45-APC-Fire750 (Clone HI30, Biolegend) and anti-human CD3-BV785 (Clone UCHT-1, Biolegend) for 1 h at 4 °C in the staining buffer (Thermo Fisher Scientific). The cells were further washed and fixed in the intracellular (IC) fixation buffer (Thermo Fisher Scientific) overnight at 4 °C. Human CD45^+^ CD3^+^ CAR-T cells were analyzed using the Attune NxT flow cytometer.

## RESULTS

The present work describes the streamlined cellular kinetics and biodistribution study for CAR-T cell therapy by ddPCR, including automated gDNA extraction procedures. The assay standardization for the cellular kinetics and biodistribution study is evaluated and discussed by incorporating the gDNA recovery study and the sample analysis in the latter section.

### The Workflow of Quantitative Cellular Kinetics and Tissue Distribution Assay by ddPCR

**Fig. 1:**
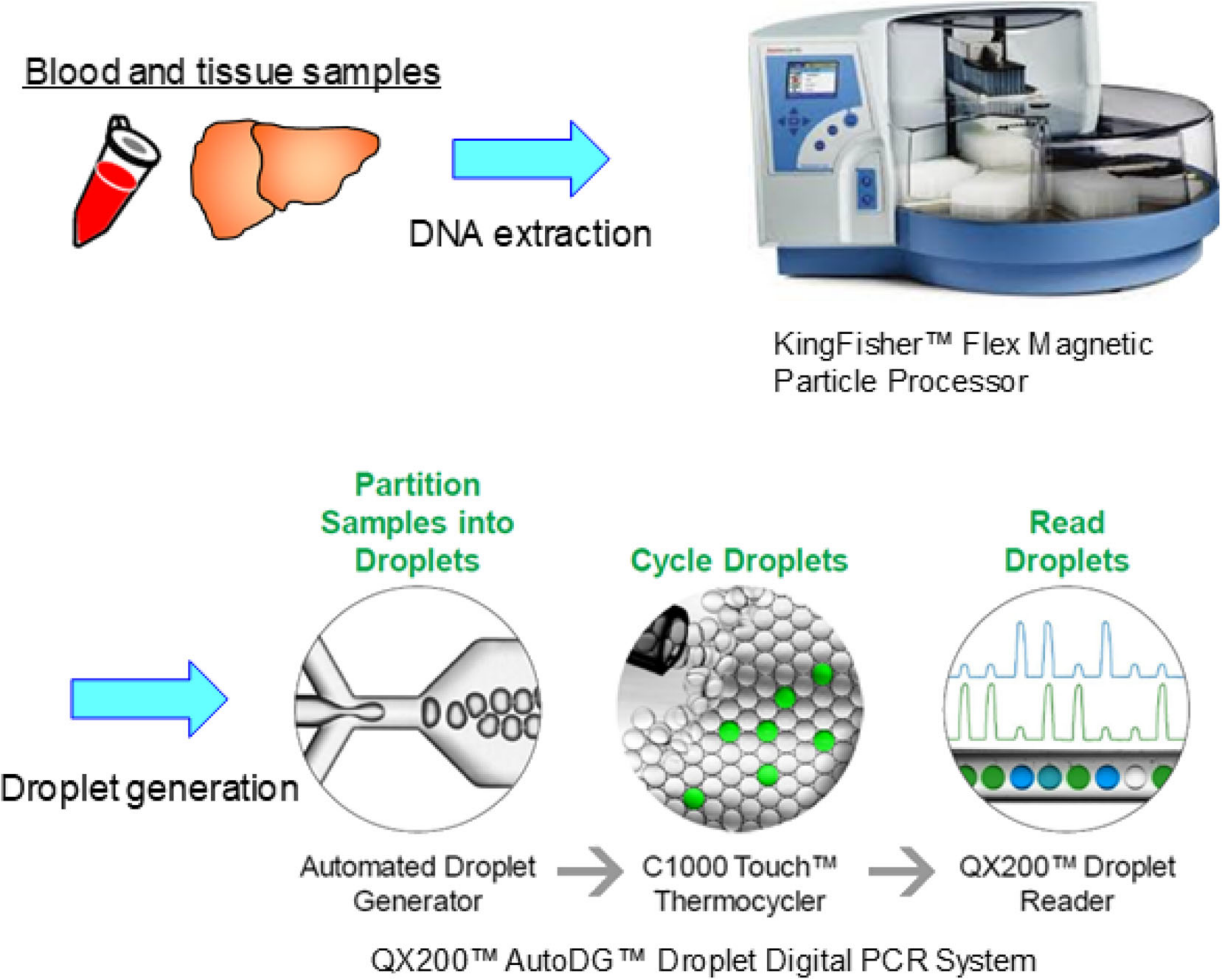
The workflow of quantitative cellular kinetics and tissue distribution assay by ddPCR

**Fig. 2:**
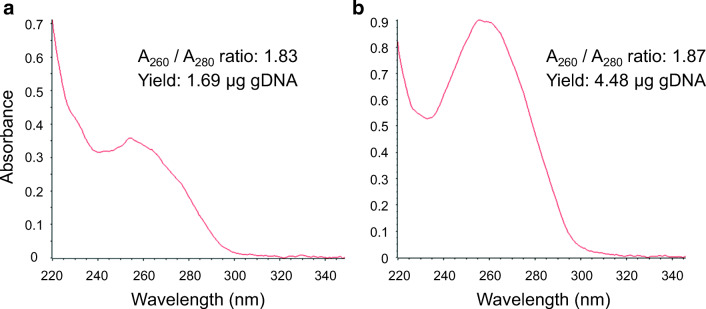
Representative UV spectra of genomic DNA prepared from NSG mouse blood. UV spectra of genomic DNA prepared from NSG mouse blood prepared by the silica filter-based manual DNA extraction (**a**) and the magnetic beads-based automated DNA extraction (**b**). UV spectra were measured over a range of wavelengths from 220 to 340 nm

A consistently good yield of high purity gDNA is required for the robust cellular kinetics and tissue distribution assay by ddPCR. We deployed the KingFisher™ Flex system assisted gDNA extraction in the presence of RNAse (combined with the tissue homogenization by gentleMACS Octo Dissociator). The extracted gDNA from mouse blood and tumor tissue showed absorbance ratios at 260 and 280 nm (A_260_/A_280_) of 1.92 ± 0.10 and 1.93 ± 0.04 (*n* = 45 each), respectively, which indicated the high purity of the extracted gDNA with minimal protein and/or RNA contamination which was further confirmed by agarose gel electrophoresis. The general workflow for cellular kinetics and tissue biodistribution assay by ddPCR is described in Fig. 1. Although the silica filter–based DNA extraction kit has been conventionally used for the gDNA extraction from blood and tissue samples, we found that the yield and purity (A_260_/A_280_ and A_260_/A_230_) of the gDNA generated by the magnetic beads–based DNA extraction procedure was consistently better than that by the silica filter-based kit, especially for NSG mouse blood sample (Fig. 2).

### Duplexing ddPCR Assay Development for Absolute Quantification of the CAR-T and Reference Gene

**Fig. 3:**
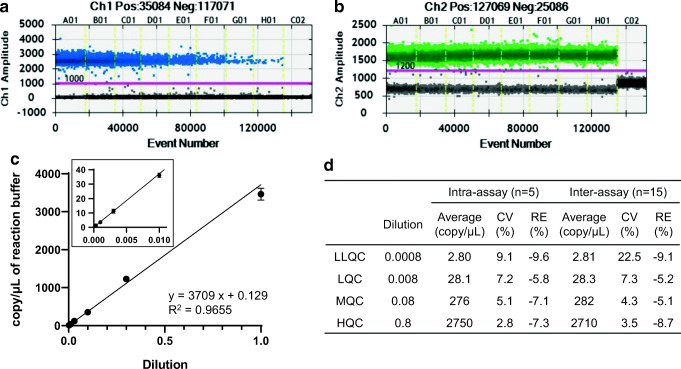
Method qualification of the CAR-T cellular kinetics assay. The ddPCR amplitude plot for the CART standard curve (A01, B01, … H01) and blank samples (C02) (**a**) and the reference gene (**b**). The standard curve for CART cells in gDNA prepared from mouse blood (**c**). The intra- and inter-assay precision and accuracy of the response in the blood QC samples (**d**)

We designed a duplexing ddPCR assay for the CAR-T transgene at the junction of CD28/CD3ζ and the reference gene (16) with detections by the 6-FAM and HEX-labeled probes, respectively. The optimized primer concentrations for the CAR-T transgene and reference gene were 100 nmol/L (Supplemental Figure 1). The optimized target-specific annealing/extension temperature in the PCR reaction was set at 63 °C for 2 min (Supplemental Figure 2). The maximum amount of the gDNA input in the ddPCR reaction was set at 200 ng of gDNA (4 μL of 50 μg/mL gDNA solution in PCR reaction) to get the reference gene copies within the dynamic range of detection. The reference gene is a target that exists as a single copy gene per haploid human genome. Therefore, 3.3 pg of gDNA contains a single copy of the reference gene. The 200 ng gDNA input in the PCR reaction generates 3030 copies/μL reaction of the reference gene (actual reference gene copy number: 3293 ± 362 copies/μL reaction, Fig. 3b), which is well below the upper limit of quantification range (5000 copies/μL) in the ddPCR system.

### ddPCR Amplitude Plot for the CAR-T Transgene Standard Curve, QC Sample Precision and Accuracy

The ddPCR amplitude plot for the CAR-T transgene and reference gene indicated that there was a clear separation between the positive (blue for the CAR-T, green for the reference gene) and negative droplets (black) with few in-between, indicating the PCR reaction is specific and efficient (Fig. 3a and b). The selectivity of the ddPCR assay for the CAR-T transgene and reference gene was assessed by measuring no template control sample (Fig. 3a and b C02). The result indicated that the contamination and primer-dimer formation are negligible. The standard curve of a series of dilutions of the CAR-T plasmid DNA in 200 ng of gDNA extracted from mouse blood was linear in the calibration range of the dilution ratios from 0.0003 to 1 (1.27–3460 copies/μL reaction; fitted with a weight factor of 1/*x*^2^ (*y* = 3709 × 0.129, *R*^2^ = 0.9655, Fig. 3c). The precision (%CV) and accuracy (%RE) in intra- and inter-assays were within 22.5% and between − 9.6 and − 5.1% of nominal values, respectively, at the dilution ratios of 0.008, 0.008, 0.08, and 0.8 from the ULOQ sample (Fig. 3d). The limit of detection (LOD) copy number was 0.35 copies/μL reaction. The volume of the PCR reaction mixture is 20 μL containing 200 ng gDNA extracted from mouse blood. Therefore, the LLOQ and LOD were 127 and 35 copy/μg gDNA, respectively. It is worth to mention that after a single intravenous administration of the CAR-T positive cells, the freeze storage stability of the mouse blood sample was confirmed at − 80 °C for at least 2 months.

### gDNA Recovery Assessment by Exo-Gene and CAR-T Cells

**Fig. 4:**
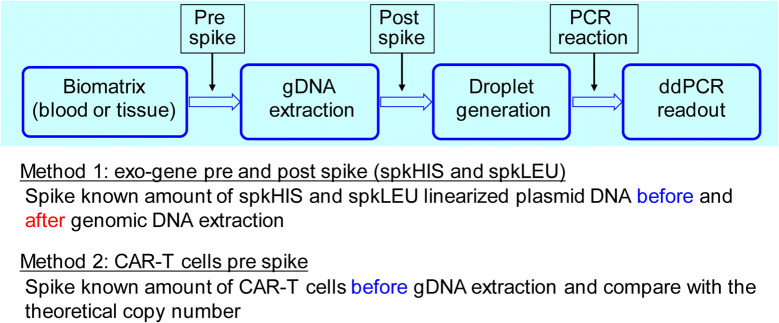
The workflow to assess the gDNA extraction recovery for unit conversion

The gDNA recovery was assessed by spiking the known amount of linearized yeast HIS3 and LEU2 plasmid DNA or the investigational CAR-T cells before the gDNA extraction process at the levels of LQC, MQC, and HQC in blood and tissues (in triplicates). The gDNA recovery was measured by comparing the measured copy numbers between the pre-spike and post-spike samples (exo-gene) or theoretical numbers (CAR-T cells). The workflow to assess the gDNA extraction recovery is described in Fig. 4. The diluted blood samples mimic the lymph depleted condition. The exo-gene recovery ranged from 60 to 100% in blood, suggesting that it may be difficult to achieve a full recovery, even with the fully automated gDNA extraction process. The reduced number of white blood cells (equivalent to the lymph depleted condition) may not significantly affect the gDNA extraction efficiency (Table I). On the other hand, the exo-gene recovery was almost quantitative from 10% liver, tumor, and kidney homogenates (Supplement Table II).

**Table I:** gDNA Extraction Recovery for Exo-Gene and CAR-T Cells

	(A)	Exo-gene HIS3 (copy/μL)				Recovery (%)	(B)	Exo-gene LEU2 (copy/μL)				Recovery (%)
		Pre-spike		Post-spike				Pre-spike		Post-spike		
		Mean	S.D.	Mean	S.D.	Mean		Mean	S.D.	Mean	S.D.	Mean
1% Mouse blood	LQC	5.4	2.0	6.9	0.5	78.4	LQC	9.9	3.0	9.0	1.1	109.6
	MQC	49.8	5.3	60.6	0.8	82.2	MQC	80.2	22.5	80.3	2.6	99.9
	HQC	430.3	21.8	540.3	12.7	79.6	HQC	635.0	104.9	752.7	27.7	84.4
5% Mouse blood	LQC	4.2	0.3	5.9	0.8	71.3	LQC	7.1	1.2	8.2	1.4	86.9
	MQC	43.1	4.4	61.7	0.5	69.8	MQC	64.0	5.0	83.5	3.8	76.7
	HQC	385.7	8.3	568.0	8.7	67.9	HQC	515.0	14.4	769.3	4.5	66.9
10% Mouse blood	LQC	4.4	0.3	6.2	0.9	70.1	LQC	5.1	1.2	7.9	0.8	63.9
	MQC	39.6	2.5	61.7	1.3	64.2	MQC	51.0	1.1	85.6	1.4	59.5
	HQC	364.7	27.7	561.0	12.0	65.0	HQC	496.7	20.8	759.3	26.4	65.4
50% Mouse blood	LQC	5.5	0.6	5.7	0.9	96.5	LQC	9.0	0.9	8.0	0.7	111.6
	MQC	59.4	2.8	62.6	1.8	94.9	MQC	78.0	5.1	81.7	2.7	95.4
	HQC	575.3	6.8	548.0	9.6	105.0	HQC	781.0	24.0	742.7	24.8	105.2
100% Mouse blood	LQC	8.0	0.9	6.9	0.5	115.5	LQC	8.9	1.0	8.3	0.6	107.7
	MQC	67.0	2.9	60.5	0.5	110.9	MQC	88.7	3.1	78.2	1.5	113.3
	HQC	601.3	26.6	579.0	13.5	103.9	HQC	837.3	18.3	798.0	28.8	104.9
	(C)	CAR-T cells (copy/μL)				Recovery (%)						
		Pre-spike		Theoretical								
		Mean	S.D.			Mean						
1% Mouse blood	LQC	12.7	1.1	13.2		96.0						
	MQC	93.5	14.8	132.2		70.7						
	HQC	1307.0	29.0	1322.3		98.8						
5% Mouse blood	LQC	10.3	1.6	13.2		78.1						
	MQC	92.8	12.1	132.2		70.2						
	HQC	772.7	58.2	1322.3		58.4						
10% Mouse blood	LQC	7.7	0.6	13.2		58.2						
	MQC	55.8	3.2	132.2		42.2						
	HQC	700.3	75.4	1322.3		53.0						
50% Mouse blood	LQC	13.8	3.0	13.2		104.4						
	MQC	135.3	18.2	132.2		102.3						
	HQC	1170.7	276.3	1322.3		88.5						
100% Mouse blood	LQC	10.2	1.0	13.2		77.4						
	MQC	145.3	4.0	132.2		109.9						
	HQC	1390.3	146.3	1322.3		105.1						

### Cellular Kinetics Data Generation with the Unit of Copy/μg gDNA and Copy/μL Blood

**Fig. 5:**
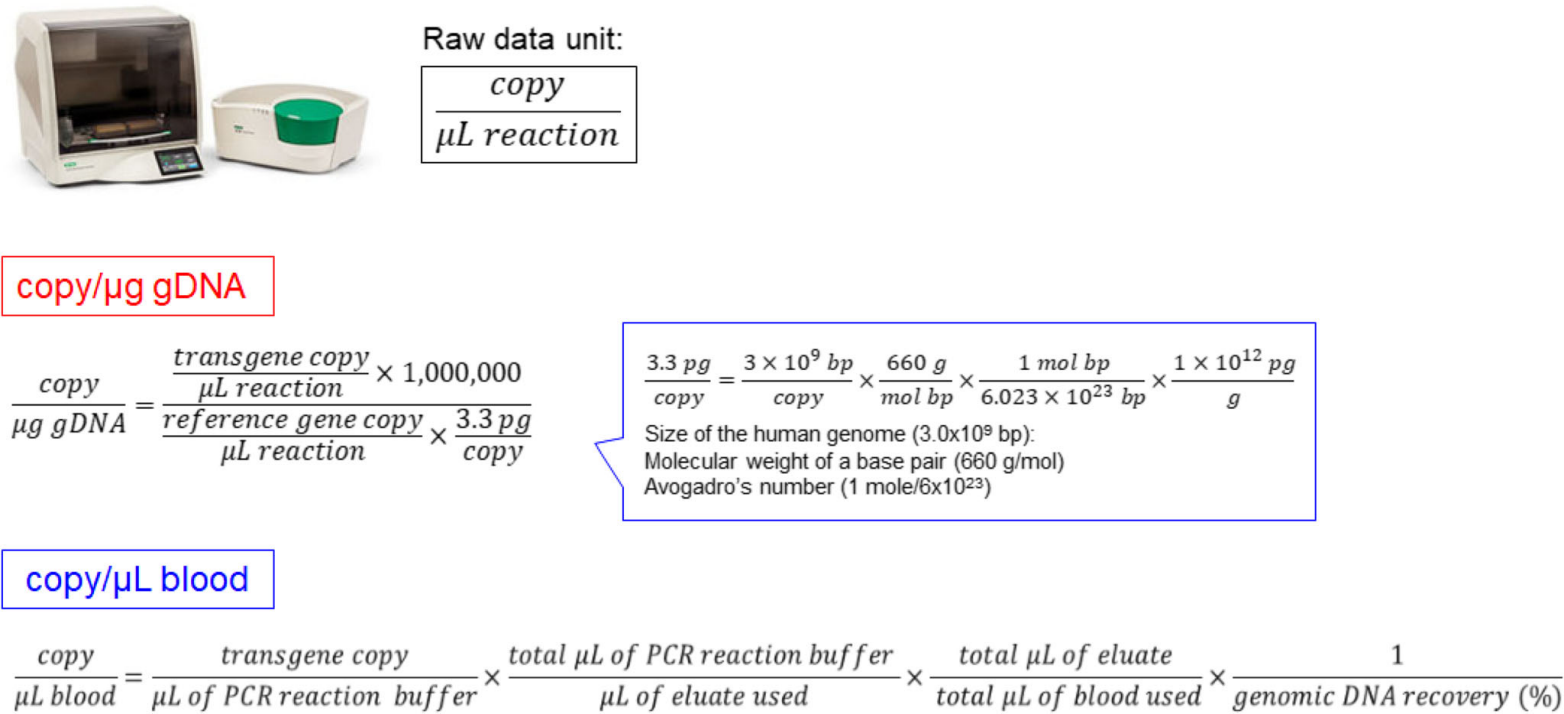
Cellular kinetics data generation by ddPCR with the unit of copy/μg gDNA and copy/μL blood. The data with a unit of **copy/μg gDNA** can be generated by the ratio of CAR-T and reference gene copy number normalized by the number of DNA per genome copy number. Although there are two copies of the target sequence in most human cells, the target sequence is present in a single copy in the human haploid genome. The haploid human genome size is 3.0 × 10^9^ base pairs. Taken into the molecular weight of a base pair (660 g/mol) and Avogadro’s number (1 mol / 6.023 × 10^23^) account, the base pair number per genome copy number can be converted to an amount of DNA per genome copy number. The data with a unit of **copy/μL blood** can be generated by the CAR-T copy number/μL PCR reaction buffer normalized by each volume such as PCR reaction buffer, gDNA elution solution, and applied blood volume as well as the gDNA recovery

The cellular kinetics data with the unit of copy/μg gDNA was generated by the CAR-T copy/μL reaction divided by the reference gene copy/μL reaction with the normalization given that 3.3 pg of human gDNA contains one copy of the target sequence. On the other hand, the cellular kinetics data with the unit of copy/μL blood was generated by the CAR-T copy number/μL PCR reaction with the normalization to the blood volume, eluate volume, and the gDNA extraction recovery. The detailed equation for generating the cellular kinetics data is summarized in Fig. 5.

### Cellular Kinetics in Female NSG Mice Bearing Human Colorectal Cancer Model

**Fig. 6:**
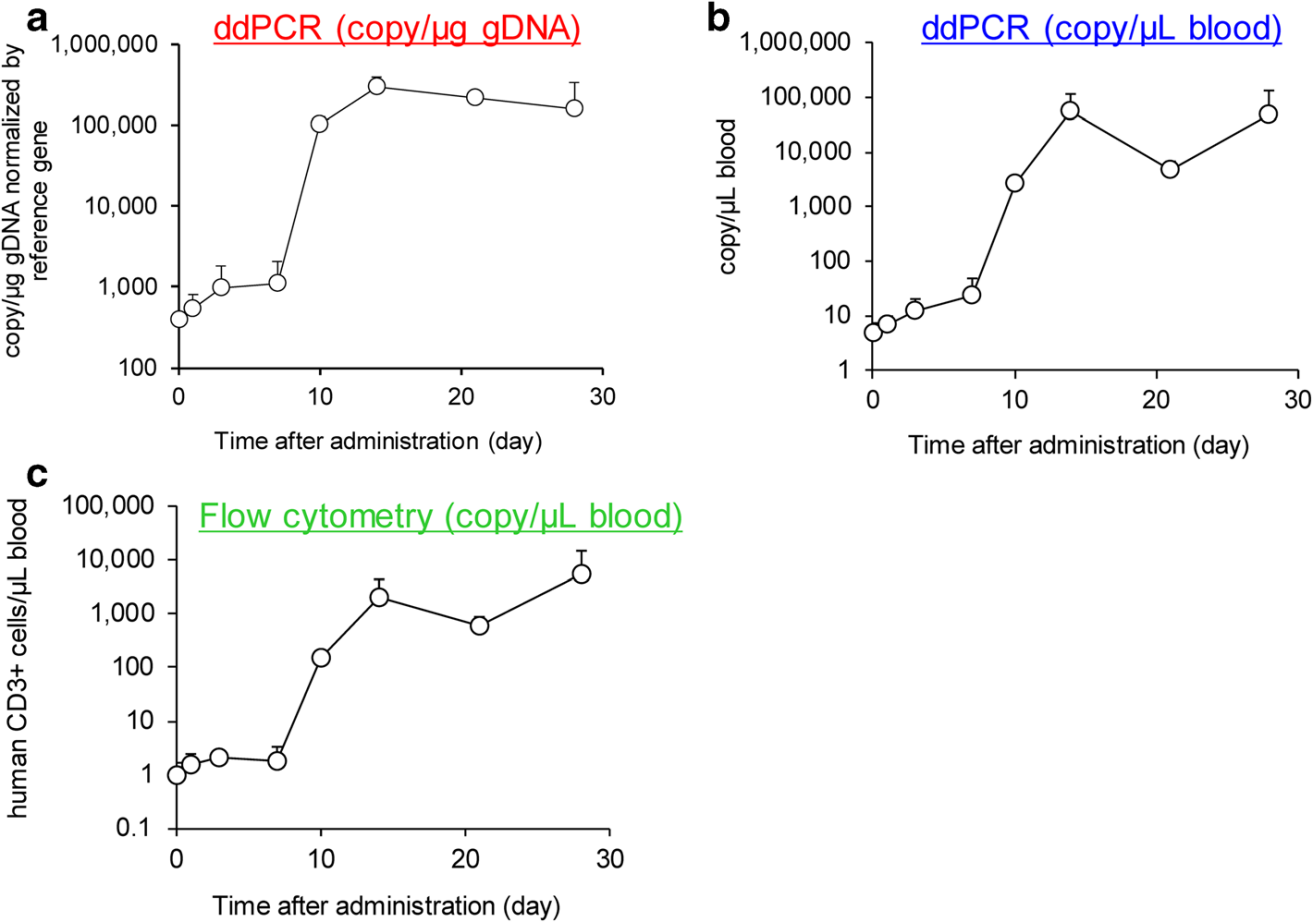
Cellular kinetics in NSG mice bearing human colorectal cancer model measured by ddPCR and flow cytometry. The time course of CAR-T transgene copy number per μg gDNA normalized by reference (**a**), per μL of blood by ddPCR (**b**), and human CD3+ CAR-T cells per μL of blood by flow cytometry (**c**). The values were expressed as mean + S.D. (*n* = 3)

The cellular kinetic study of CAR-T cells in mouse blood was conducted by ddPCR (Fig. 6a and b) and flow cytometry (Fig. 6c) after a single intravenous administration of CAR-T positive cells at a dose of 1.0 × 10^6^ cells/mouse. Immediately after the administration, the CAR-T transgene level appeared very low because of the quick distribution of cells throughout the peripheral blood, bone marrow, lung, and other tissues, which is comparable with the previous report (11). Seven days after the administration, the rapid *in vivo* expansion of CAR-T cells in blood was observed in both ddPCR and flow cytometry assays. The cellular kinetics profile of CAR-T cell expansion was well correlated between the transgene level by ddPCR and the CAR-T expression level by flow cytometry. The magnitude of CAR-T cell expansion was comparable between ddPCR with the unit of copy/μL blood (Fig. 6b) and the flow cytometry-based assay (Fig. 6c). However, the transgene level with the unit of copy/μg gDNA (Fig. 6a) notably underestimated the CAR-T cell expansion compared with other methods.

## DISCUSSION

The expansion and persistence of transduced CAR-T cells in the blood and targeted tumor tissue are two important factors to show the antitumor efficacy of immunotherapy in cancer therapy. The recent FDA guidance for industry recommended that the sensitive and quantitative analytical methods detect the transgene sequences in the preclinical biodistribution study by quantitative PCR assays (7). The conventional qPCR can provide an absolute quantification of targeted transgene based on calibration curves prepared by a known amount of a synthetic external transgene calibrator in a sample matrix (17). On the contrary, ddPCR is an emerging technology uniquely characteristic of the partitioning the target gene analyte into approximately 20,000 of 1 nL size droplets (10). The distribution of the transgene target analyte in the partitioned sample is calculated by Poisson distribution and can be quantified without an external calibrator. Thus, a ddPCR-based assay can potentially overcome the inaccuracy of calibration and normalization needed in a qPCR-based assay. A general comparison between qPCR and ddPCR is summarized in Table II.

**Table II:** A Comparison Between Quantitative PCR and Droplet Digital PCR

	Quantitative PCR	Droplet digital PCR
Calibration curve	External calibration	Not required for absolute quantification
Precision	Relatively larger variations	Consistently shown lower variations
Sensitivity	Comparable limit of quantification	
Quantification	Rely on amplification efficiency	End-point approach (more tolerant to PCR inhibitor)
Duplexing assay	FAM / HEX (VIC) Wider calibration ranges	FAM / HEX (VIC) Narrower calibration ranges

*The ddPCR assays use the end-point analysis to generate quantitative data. Therefore, the amplification efficiency plays a smaller role than that in quantitative PCR*

As a new technology, a fit for purpose method qualification for the transgene copy number by ddPCR may increase the confidence in their usefulness and reliability of the assay technology. Although there is no official validation guidance currently available for the ddPCR-based assay to be applied for sensitive gene expression analysis, the qualification parameters such as selectivity, calibration curve, quality control samples, gDNA recovery, and stability of the analyte in the matrix may be evaluated based on the general principles in bioanalytical method validation guidance for industry published by FDA (18). Due to the unique characteristics of ddPCR for the absolute quantification of nucleic acid target sequences without the requirement of an external calibrator or endogenous control, the calibration curve and QC samples were prepared from a serial dilution of CAR-T plasmid DNA in gDNA extracted from mouse blood. The current assay satisfied the qualification acceptance criteria in terms of selectivity, linearity of the calibration curve, precision and accuracy of QC samples, and sample stability (Fig. 3).

The standardization of the cellular kinetics and biodistribution assay to characterize the CAR-T cell distribution, expansion, contraction, and persistence (DECP) profile becomes “the whole industry interest” to support the cell therapy. For a related therapy, the recent FDA guidance for preclinical study design to assess biodistribution and persistence of gene therapy products recommended that the bioanalysis be conducted by a quantitative PCR assay capable of detecting vector sequence in animal and human tissues. In addition, the assay should have a demonstrated limit of quantitation of 50 copies/μg genomic DNA (7). The advantage of this specifically referred reporting unit attributes to normalize the gDNA extraction efficiency and individual difference in gDNA content in biomatrix samples (19). However, when CAR-T cells expand in blood and tissue, the apparent transgene copy number with the copy/μg gDNA unit may underestimate the CAR-T cell expansion since the gDNA content of a blood or tissue sample is increasingly contributed from the expanded CAR-T cells. On the contrary, the transgene copy number normalized by the associated blood volume or tissue weight (copy/μL blood or mg tissue) is independent of the gDNA content of a sample and thus is not subject to the influence of the expansion status of the CAR-T cells. Therefore, we integrated the gDNA recovery assessment (Fig. 4, Table I, Supplement Table II) along with the sample analysis, the cellular kinetics, and biodistribution data and were able to generate data in both FDA regulatory compliant copy/μg gDNA unit and the copy/μL blood or mg tissue unit for their comparison (Figs. 5 and 6).

The cellular kinetics data in female NSG mice bearing human colorectal cancer xenografts after a single intravenous administration of CAR-T positive cells indicated that there was a higher correlation between the transgene level with the unit of copy/μL blood by ddPCR and the cell surface total CAR-T expression by flow cytometry (Fig. 6b and c). Although the trend of cellular kinetics determined by ddPCR and flow cytometry matched well, the possible reasons when it lacks concordance in terms of the absolute copy number are that multiple copies of the transgene may be inserted per cell, silencing of the transgene, and intracellular trapping or downregulation of CAR expression which may cause the higher copy number detected by ddPCR than that by flow cytometry (20, 21). From a sample procurement perspective, the ddPCR-based cellular kinetics assay has a unique advantage since any biomatrix samples, including the solid tumor, can be stored frozen until the analysis. Conversely, the samples need to be processed and analyzed within a few days for flow cytometry–based assay. Nevertheless, the flow cytometry–based assay can characterize CAR-T cells and detection for multiple immunological cell markers. In this study, we have only measured the total CD3^+^ T cell population rather than CAR-T positive cells due to the availability of antigen-specific antibodies.

**Fig. 7:**
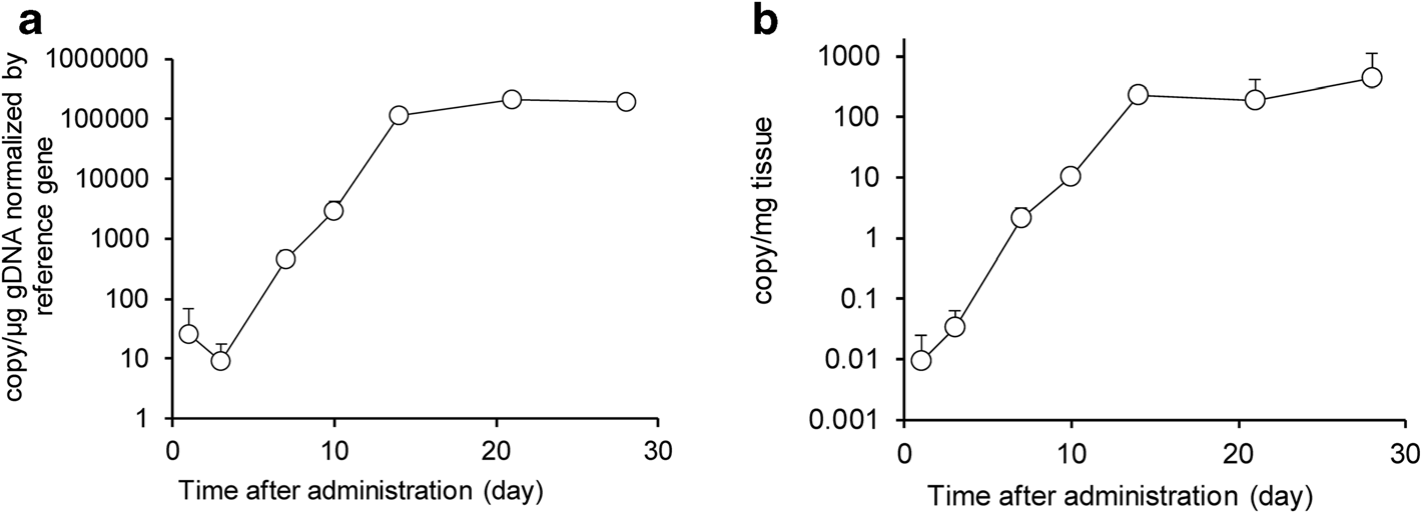
Tumor distribution of CAR-T cells in NSG mice bearing human colorectal cancer model measured by ddPCR. The time course of CAR-T transgene copy number per μg gDNA normalized by reference (**a**) and per mg tissue (**b**) by ddPCR. The values were expressed as mean + S.D. (*n* = 3)

The most striking difference between the conventional small molecule or antibody-based biotherapeutics and CAR-T cell therapy is that CAR-T cells can expand and proliferate *in vivo*. CAR-T cells are known to distribute rapidly to the tissue within a few hours after the intravenous administration (22). After 7–10 days of the administration, the copy number of CAR-T transgene exponentially increased and reached the Cmax within 2 weeks, followed by a contraction and persistence phase indicating that the investigational CAR-T cells persisted well in the systemic circulation (Fig. 6). The elimination of CAR-T cells was reported to be much faster in the lungs due to the presence of macrophages via apoptosis (23). The biodistribution to the tumor tissue has a similar trend in cellular kinetic profiles. After expansion, the CAR-T transgene level was maintained for at least 28 days post-administration because of the specific target expression on the solid tumor cell surface (Fig. 7). This result suggested that the investigational CAR-T cells have a huge potential to overcome insufficient or slow penetration of CAR-T cells in the solid malignancies, which is one of the challenges of developing a CAR-T cell therapy for solid tumors.

## OPPORTUNITIES, CHALLENGES, LIMITATIONS, AND FUTURE OUTLOOK ON ddPCR-BASED CELLULAR KINETICS AND BIODISTRIBUTION ASSAY

In the drug discovery and development phase of *in vivo* and *ex vivo* gene therapy, cellular kinetics and biodistribution have become an integral part of efficacy and safety assessment in nonclinical and clinical studies (24). The emergence of the ddPCR technology over the past ten years has expanded the opportunity and potential for its application toward the innovative *in vivo* and *ex vivo* gene therapy regardless of therapeutic areas (i.e., immuno-oncology cell therapy and gene therapy in rare diseases). However, the ddPCR technique also faces multiple sizable challenges that need to be addressed for becoming the mainstream for *in vivo* and *ex vivo* gene therapy support. Herein, we discussed the potential opportunities and limitations of the ddPCR-based cellular kinetics and biodistribution assay platform in the best practical way to implement this platform from the pharmaceutical industry perspective.

The unique characteristic of partitioning based on Poisson statistics made the absolute quantification possible by digital PCR (25, 26). The partitioning can be performed either by the physically isolated chambers (9) or water-in-oil droplet emulsion (27). However, the conventional qPCR-based assay requires the calibration curve to define the relationship between dilutions of the target and the threshold cycle (C_T_) values. The copy numbers of nucleic acids determined by UV measurement tend to be inaccurate due to the contamination of impurities (Supplemental Figure 3). If the stock concentration of reference standard is defined by the UV measurement, there is a risk of overestimating of the actual sample concentration. Besides, the generation of calibration curve requires significant dilution (>5 logarithmic range) with multiple replication at each concentration that may make inter-laboratory comparison challenging.

The standardization of ddPCR-based cellular kinetics and biodistribution assay with the current regulatory compliant unit (copy/μg gDNA) and the pharmacologically meaningful unit (copy/μL blood or copy/mg tissue) for the subsequent mechanistic modeling work are required to characterize the *in vivo* expansion of CAR-T transgene (13). Fehse *et al*. first reported that the cellular kinetics assay of CD19-CAR-T cells with copy/μL blood unit by combining white blood cell (WBC) count to normalize the gDNA extraction efficiency (12). Although this assay format can be applicable for both nonclinical and clinical studies, this approach’s limitation is that the mean copy number of CAR-T cells with a specific transduction rate needs to be assumed because the transduction rate can be variable depending on the *in vivo* CAR-T cell activation. Besides, the quality of WBC counting data especially in the lymphodepletion condition may be inaccurate. Yamamoto *et al*. reported the qPCR-based cellular kinetics assay with copy/μL blood unit and its comparison with the CAR+ cell number determined by flow cytometry (13). Their method incorporates the spike-in calibration curve with an external control gene (dog gDNA) to normalize the variability. To our best knowledge, our proposed assay reported in the manuscript is the first one applicable for both cellular kinetics and biodistribution of CAR-T cell therapy. The automated gDNA extraction procedure enabled higher yield and better reproducibility of gDNA extraction than the conventional labor-intensive manual extraction (Fig. 2). The multiplexing capability of CAR-T and reference gene detection made an accurate normalization of the gDNA input for each PCR reaction.

The primary advantage of absolute quantification by ddPCR brings a new challenge of the definition of the nominal concentration. In the conventional bioanalysis, the nominal concentration is defined by the standard curve, which is prepared by the serial dilution of a reference standard stock solution. Whether the absolute quantification by ddPCR or the concentration by UV measurement is defined as the nominal concentration is similar to the “chicken or egg” paradox. Based on our experience and experimental data shown in Supplemental Figure 3, the nucleic acid concentration by UV absorbance may overestimate the actual concentrations due to the contaminants such as salts, organic solvents, detergents, and proteins. Fluorescent-based dyes that emit fluorescence when bound to the targeted nucleic acid (Qubit™ and Quant-iT™ PicoGreen™) provide more sensitive and accurate quantification results that are closer to the concentration measured by ddPCR.

The second challenge is the potential variability of the droplet’s quality (number and volume) generated by the QX200™ Droplet Generator. Although a PCR reaction mixture can be theoretically partitioned up to 20,000 droplets, the droplet’s actual number during the method qualification was 16,048 ± 1495 (Fig. 3). Pinheiro *et al*. reported that when the number of partitioning is more than 10,000, the copy number measurement of the target gene can be achieved with a very high precision level (26). Another potential cause of uncertainly is associated with the droplet volume as the droplet volume is defined as 0.85 nL in the latest software version from Bio-Rad. Corbisier *et al*. determined the average droplet volume by optical microscopy and the measured volume (0.834 nL) is very close to the defined volume in QuantaSoft analysis software version 1.7 (28). The wrong size, shape, and clustered droplets are excluded for the copy number measurement. Therefore, the impact of excluded droplets on the analytical data should be negligible.

The third challenge is the potential variability of gDNA recovery, especially in the blood sample with the reduced number of white blood cells in the lymphodepletion condition in the clinic. The gDNA recovery in 100% blood and 10% liver, tumor, and kidney homogenates was almost quantitative. However, the lower recovery was observed in the diluted blood samples, which mimic the lymphodepletion condition (Table I and Supplement Table II). Although the normalization of gDNA recovery in the individual sample is feasible, this may add additional costs and labor to generate the cellular kinetics and biodistribution of CAR-T cells with the volume-based unit. Further improvement of an automated gDNA extraction may need to be investigated.

The ddPCR is a technology equally new to the pharmaceutical industry and the regulatory agency. At this moment, there is no regulatory guidance available regarding the ddPCR-based cellular kinetics and biodistribution assay for CAR-T cell therapy. Therefore, the fit-for-purpose approach based on the currently existing bioanalytical method validation guidance for the industry would be an appropriate strategy (Fig. 3). The accumulation of method qualification and validation data to establish the practical and scientifically underpinning acceptable criteria for the assay assessment is preferred. The minimum method qualification parameters (run in triplicate) may include selectivity and specificity (non-template control, NTC), linearity (calibration curve prepared by the spike-in standard of the linearized plasmid into the gDNA solution), precision and accuracy (LQC, MQC, and HQC), sensitivity, dilution linearity, and recovery of target nucleotides. An appropriate short- and long-term stability test may be warranted in method qualification. Furthermore, the inter-laboratory cross-validation will ensure the reliability of the same ddPCR-based assay performed in multiple laboratories for the CAR-T transgene and reference gene detection. As more ddPCR-based assay is being performed to support cellular kinetics and biodistribution assessment and more experience, including method qualification and validation are accumulated from both industry and regulatory perspective, it is foreseeable that in-depth insights on the technology will be shared within the bioanalytical community between the industries and regulators. This will further facilitate the advancement and adoption of the technology. In the authors’ opinion, the ddPCR-based genomic assay will likely play a critical role in the cell and gene therapy field in terms of transgene quantification using high-throughput and user-friendly systems that may eventually fundamentally change the industry practice in the genomic assay domain.

## CONCLUSION

We developed a novel strategy for assessing the cellular kinetics and biodistribution by using an emerging ddPCR platform. By incorporating the automated gDNA extraction process and gDNA recovery test along with sample analysis, the newly established assay schemes for cellular kinetics and biodistribution assay met both the current regulatory requirements and subsequent pharmacokinetic analysis in a single bioanalytical run. Our findings may provide insights on the ddPCR-based cellular kinetics and biodistribution assay for the CAR-T cell therapy as a potential standardized approach.

## Supplementary Information

ESM 1 (DOCX 452 kb)
