# Supplementary material for: Insights on Droplet Digital PCR–Based Cellular Kinetics and Biodistribution Assay Support for CAR-T Cell Therapy
Source: AAPS J. 2021 Mar 2;23(2):36. doi: 10.1208/s12248-021-00560-6 (PMC7925486; doi:10.1208/s12248-021-00560-6)
Supplement: Supplementary file 1 — (DOCX 452 kb) [file 12248_2021_560_MOESM1_ESM.docx]

**Supporting Information**

**Insights on droplet digital PCR based cellular kinetics and biodistribution assay support for CAR-T cell therapy**

Hiroshi Sugimoto^1*^, Susan Chen^2^, Jean-Pierre Minembe^2^, Johara Chouitar^3^, Xingyue He^3^, Haiqing Wang^2^, Xiaodong Fang^2*^, Mark G. Qian^2^

^1^ Department of Drug Metabolism and Pharmacokinetics, Takeda Pharmaceuticals International Co, 125 Binney Street, Cambridge, MA 02142, USA

^2^ Department of Drug Metabolism and Pharmacokinetics, Takeda Pharmaceuticals International Co, 35 Landsdowne Street, Cambridge, MA 02139, USA

^3^ Department of Immuno Oncology DDU, Takeda Pharmaceuticals International Co,

40 Landsdowne Street, Cambridge, MA 02139, USA

Supporting information contents

Supplemental Figure 1:

Optimization of the duplexing capability of CAR-T transgene and reference gene detection by ddPCR

Supplemental Figure 2:

Optimized annealing/extension temperature in the PCR protocol for CAR-T

Supplemental Figure 3:

Comparison of gDNA concentration using UV measurement by spectrophotometer and reference gene detection by ddPCR

Supplemental Table I:

Primers and probes used for CAR-T transgene and yeast exo-gene detection

Supplement Table II:

gDNA extraction recovery for exo-gene in tissue

*Optimization of the duplexing capability of CAR-T transgene and reference gene detection by ddPCR*

The optimal condition for reference gene primer concentrations (10, 20, 50, 100, and 900 nmol/L) was investigated for duplexing assay of CAR-T transgene and reference gene detection by ddPCR. In the presence of 100 nmol/L of each primer, the positive and negative signal is well separated. A lower concentration of primer for reference gene decreased the quality of separation and a higher concentration of each primer interfered with the PCR efficiency (Supplemental Figure 1). The annealing temperature was optimized by carrying out temperature gradient PCR for both CAR-T transgene and reference gene (Supplemental Figure 2).

Supplemental Figure 1: Optimization of duplexing capability of CAR-T transgene and reference detection by ddPCR

Supplemental Figure 2: Optimized annealing/extension temperature in the PCR protocol

*Comparison of gDNA concentration* *using UV measurement by spectrophotometer and reference gene detection by ddPCR*

Reliable and robust measurement of gDNA concentration is the foundation for PCR-based assay. gDNA concentration was measured using UV measurement by spectrophotometer and reference gene detection by ddPCR. The UV absorbance is converted into μg/mL of double-stranded DNA (dsDNA) using the established conversion factor of 50 at 260 nm. The ddPCR-based reference gene copy number/μL is converted into μg/mL of gDNA using above mentioned equation of 3.3 pg/single copy. Although gDNA determined by UV measurement and reference gene generally matched quite well, however, the number of gDNA determined by UV measurement overestimated compared to that by reference gene due to the potential contamination of RNA or organic solvent residue in the eluate, especially early time point after CAR-T administration (Supplemental Figure 3).

Supplemental Figure 3: Comparison of gDNA concentration using UV measurement by spectrophotometer and reference gene detection by ddPCR (A). The time course of CAR-T transgene copy number per µg gDNA normalized by reference (open black circle) and spectrophotometer (open red circle) by ddPCR (B) The values were expressed as mean+S.D. (n=3).

Supplemental Table I:

Primers and probes used for CAR-T transgene and yeast exo-gene detection

| Target (gene) | Primer/probe | Sequence (5’-3’) | Final concentration  (nmol/L) |
| --- | --- | --- | --- |
|  | CD28 F1 | CCCACCCGCAAGCATTACCA | 100 |
| CAR-T | CD3ζ R1 | GGTTCTGGCCCTGCTGGT | 100 |
|  | CAR-T probe | /56-FAM/TCGCTCCAG/ZEN/AGTG  AAGTTCAGCAGGAGC/3IABkFQ/ | 100 |

| Target (gene) | Primer/probe | Sequence (5’-3’) | Final concentration  (nmol/L) |
| --- | --- | --- | --- |
|  | HIS3 F1 | CGCAAATCCTGATCCAACC | 100 |
| HIS3 | HIS3 R1 | ACTGAAGACTGCGGGATTG | 100 |
|  | HIS3 probe | /56-FAM/AGGGCCTCT/ZEN/ TTAAAAGCTTGACCGA/3IABkFQ/ | 100 |
| LEU2 | LEU2 F1 | TGCTAAAGGTACTGACTTCGTTG | 100 |
|  | LEU2 R1 | GATTCTTTGCACTTATGGAACG | 100 |
|  | LEU2 probe | /5HEX/ATGGTGATG/ZEN/ GTGTCACTTGGGA/3IABkFQ/ | 100 |

Supplement Table II: gDNA extraction recovery for exo-gene in tissue

|  | (A) | Exo-gene HIS3 (copy/µL) | | | | Recovery  (%) |  | (B) | Exo-gene LEU2 (copy/µL) | | | | Recovery  (%) |  |
| --- | --- | --- | --- | --- | --- | --- | --- | --- | --- | --- | --- | --- | --- | --- |
|  |  | Pre-spike | | Post spike | |  |  |  | Pre-spike | | Post spike | |  |  |
|  |  | Mean | S.D. | Mean | S.D. | Mean |  |  | Mean | S.D. | Mean | S.D. | Mean |  |
| 10% Liver homogenate | LQC | 6.9 | 0.9 | 7.0 | 0.2 | 98.6 |  | LQC | 9.3 | 0.7 | 8.6 | 0.7 | 107.2 |  |
|  | MQC | 68.1 | 5.6 | 65.9 | 1.5 | 103.3 |  | MQC | 89.3 | 3.6 | 87.3 | 4.0 | 102.3 |  |
|  | HQC | 668.0 | 21.2 | 677.3 | 23.6 | 98.6 |  | HQC | 889.0 | 24.6 | 894.0 | 23.5 | 99.4 |  |
| 10% Tumor homogenate | LQC | 7.2 | 0.4 | 6.6 | 1.0 | 108.3 |  | LQC | 9.1 | 0.8 | 9.1 | 0.6 | 100.3 |  |
|  | MQC | 65.3 | 5.1 | 66.3 | 2.9 | 98.6 |  | MQC | 85.4 | 7.3 | 84.6 | 2.1 | 100.9 |  |
|  | HQC | 641.3 | 50.5 | 669.5 | 15.6 | 95.8 |  | HQC | 861.5 | 79.8 | 880.3 | 12.8 | 97.9 |  |
| 10% Kidney homogenate | LQC | 7.6 | 0.6 | 7.4 | 1.3 | 103.1 |  | LQC | 9.4 | 0.9 | 8.6 | 0.7 | 109.9 |  |
|  | MQC | 71.8 | 5.0 | 72.3 | 1.5 | 99.3 |  | MQC | 93.5 | 5.8 | 90.3 | 2.1 | 103.6 |  |
|  | HQC | 686.0 | 19.4 | 684.3 | 6.2 | 100.3 |  | HQC | 940.0 | 49.6 | 902.5 | 21.2 | 104.2 |  |
